# Compositional differences in gastrointestinal microbiota in prostate cancer patients treated with androgen axis-targeted therapies

**DOI:** 10.1038/s41391-018-0061-x

**Published:** 2018-07-09

**Authors:** Karen S. Sfanos, Mark C. Markowski, Lauren B. Peiffer, Sarah E. Ernst, James R. White, Kenneth J. Pienta, Emmanuel S. Antonarakis, Ashley E. Ross

**Affiliations:** 1 Department of Pathology, Johns Hopkins University School of Medicine, Baltimore, MD USA; 2 Sidney Kimmel Comprehensive Cancer Center, Baltimore, MD USA; 3 Department of Urology, James Buchanan Brady Urological Institute, Johns Hopkins University School of Medicine, Baltimore, MD USA; 4 Department of Molecular and Comparative Pathobiology, Johns Hopkins University School of Medicine, Baltimore, MD USA; 5 Resphera Biosciences, Baltimore, MD USA; 6 Present Address: Texas Urology Specialists, Dallas, TX USA

## Abstract

**Background:**

It is well known that the gastrointestinal (GI) microbiota can influence the metabolism, pharmacokinetics, and toxicity of cancer therapies. Conversely, the effect of cancer treatments on the composition of the GI microbiota is poorly understood. We hypothesized that oral androgen receptor axis-targeted therapies (ATT), including bicalutamide, enzalutamide, and abiraterone acetate, may be associated with compositional differences in the GI microbiota.

**Methods:**

We profiled the fecal microbiota in a cross-sectional study of 30 patients that included healthy male volunteers and men with different clinical states of prostate cancer (i.e., localized, biochemically recurrent, and metastatic disease) using 16S rDNA amplicon sequencing. Functional inference of identified taxa was performed using PICRUSt.

**Results:**

We report a significant difference in alpha diversity in GI microbiota among men with versus without a prostate cancer diagnosis. Further analysis identified significant compositional differences in the GI microbiota of men taking ATT, including a greater abundance of species previously linked to response to anti-PD-1 immunotherapy such as *Akkermansia muciniphila* and *Ruminococcaceae* spp. In functional analyses, we found an enriched representation of bacterial gene pathways involved in steroid biosynthesis and steroid hormone biosynthesis in the fecal microbiota of men taking oral ATT.

**Conclusions:**

There are measurable differences in the GI microbiota of men receiving oral ATT. We speculate that oral hormonal therapies for prostate cancer may alter the GI microbiota, influence clinical responses to ATT, and/or potentially modulate the antitumor effects of future therapies including immunotherapy. Given our findings, larger, longitudinal studies are warranted.

## Introduction

The gastrointestinal (GI) microbiota are known to influence the metabolism, pharmacokinetics, and toxicity of many drugs and xenobiotics [1], yet there are few mechanistic studies exploring this effect in relation to cancer therapies. Several compelling examples have emerged providing insight into the relationship between human-associated microbiota and cancer treatment. The bacterium *Mycoplasma hyorhinis* and species of *Proteobacteria*, when present within a tumor, may metabolize the chemotherapy drug, gemcitabine, into a deaminated inactive metabolite [2], resulting in drug resistance [3]. β-glucuronidases produced by bacterial species in the GI tract can reactivate the excreted, inactive metabolite of the topoisomerase I inhibitor, irinotecan, causing adverse drug toxicities, including severe diarrhea [4]. Likewise, although the mechanism is not fully understood, there is emerging evidence that the GI microbiota can influence the efficacy of immunotherapy [5–11].

Recent studies in animal models have demonstrated that intestinal microbiota are essential for therapeutic efficacy of agents such as cyclophosphamide [7], platinum chemotherapy [6], and both anti-CTLA-4 [5] and anti-PD-L1 [8] immunotherapies. Eradication of the commensal intestinal flora by antibiotic treatment or via use of germ-free mice eliminates therapeutic efficacy of these agents in different tumor models. A study in a melanoma model showed that the therapeutic benefit of anti-PD-L1 immunotherapy could be bolstered by feeding animals a strain of *Bifidobacterium*—a species commonly used in probiotic supplements—prior to initiating therapy [8]. Three recent human studies, two in melanoma patients [9, 11] and one in patients with epithelial tumors [10], reported that the presence of certain types of bacteria including *Ruminococcaceae, Bifidobacteriaceae*, and *Akkermansia muciniphila* are associated with response to anti-PD-1 immunotherapy. Fecal microbial transplant from human donors that were responders to anti-PD-1 immunotherapy into germ-free mouse allograft tumor models conferred antitumor efficacy of anti-PD-1 immunotherapy versus fecal samples transplanted from non-responders [9–11]. Collectively, these studies indicate that members of the intestinal microbiome may be essential for cancer drug efficacy and that modulating intestinal microbiome composition may enhance therapeutic response.

The relationship between the GI microbiota and cancer therapies in men with prostate cancer is underexplored. There is, however, compelling evidence that the GI microbiome is involved in multiple-related processes such as modulation of circulating hormone levels [12, 13], stimulation of antitumor immune responses [5, 6, 8], and induction of treatment-related toxicities (including immunotherapy-induced colitis [14] and radiation-induced bowel toxicity [15]), and/or morbidities including development of metabolic syndrome [16, 17]. Animal studies suggest that the GI microbiota may also be affected by circulating androgen levels [12, 13] and castration [16]. We hypothesize that hormonal therapy, particularly oral formulations of androgen axis-targeted drugs, used in the treatment of prostate cancer may promote changes in the GI microbiota. In this exploratory study, we determined compositional differences in GI microbiota in (1) men with and without prostate cancer and (2) men with localized prostate cancer, biochemical recurrence after primary treatment, and hormone-sensitive or castration-resistant metastatic disease. We also examined the relationship between GI microbiota composition and androgen deprivation therapies, with a focus on orally administered androgen receptor axis-targeted therapies (ATT).

## Patients and methods

### Study design and patient population

Specimens were obtained under a Johns Hopkins Medicine Institutional Review Board approved protocol with written informed consent. Rectal swabs from 30 patients were collected during routine Urology or Medical Oncology outpatient clinic visits at the Johns Hopkins Hospital and Sydney Kimmel Comprehensive Cancer Center. Patients who were currently taking an antibiotic were excluded. Patients designated as “controls” were being followed in the Urology clinic primarily for benign prostatic hyperplasia. Importantly, since the control patients did not undergo prostate biopsy, they cannot be definitively defined as cancer free. Men categorized as “benign” were being evaluated for suspicion of prostate cancer, but subsequently had a negative biopsy. For the benign group, the rectal swab was collected at the evaluation clinic visit, which was prior to the patient taking prophylactic antibiotics for the biopsy. For the seven men in the “cancer” group, three had swabs taken 1–2 months prior to their diagnostic biopsy, one had their swab taken 1 month after diagnostic biopsy, and three men had swabs taken >6 months after prior biopsy. For the purposes of our medication analyses, the designation “NoMeds” indicated men who were not undergoing androgen derivation therapy (ADT), “GNRH” were men only being treated with a gonadotropin-releasing hormone (GNRH) agonist/antagonist, and “oral ATT” were men being treated with oral androgen receptor axis-targeted therapies.

### Sample collection and DNA isolation

The rectal swab procedure is detailed in the Supplemental Methods. Rectal swabs were immediately stored at −80 °C until DNA isolation. The investigators were blinded to group allocation until after all sequencing was completed. Swab contents were resuspended in 500 μl of 1× PBS and DNA was extracted with a phenol:chloroform method that incorporates multiple enzyme digest and bead beating as previously described [18]. A total of 16 “mock” (500 μl 1× PBS as starting material) DNA extractions were performed to control for contamination from DNA extraction through the full amplification and sequencing pipeline.

### 16S rDNA gene library generation, HiSeq sequencing, and analysis

Details of the sequencing and analysis can be found in the Supplemental Methods.

### Statistical analysis

After contaminant removal, random subsampling to 80,000 sequences per sample was performed to provide even coverage prior to downstream statistical comparisons (rationale for subsampling described in ref. [19]). Differential abundance analysis was performed using the negative binomial test implemented in the DESeq R package. *P* values were adjusted for multiple hypothesis testing using the false discovery rate (FDR). Beta diversity analysis, including Bray-Curtis and UniFrac distance computation and principal coordinates analysis (PCoA), was performed in QIIME. Statistical comparisons of alpha diversity utilized generalized linear models (GLMs) and evaluated three different underlying response variable family distributions (Gaussian, Log-Normal, and Gamma).

### *Akkermansia muciniphila* quantitative PCR

*A. muciniphila* genomic DNA was obtained from the American Type Culture Collection (BAA-835D-5) to develop a standard curve for quantitative PCR (qPCR). The *A. muciniphila* species-specific primer set was used as follows, Forward primer: 5′-CAGCACGTGAAGGTGGGGAC-3′, Reverse primer: 5′-CCTTGCGGTTGGCTTCAGAT-3′. The total estimated copies of *A. muciniphila* in each fecal sample were determined using this assay relative to the total number of estimated 16S rDNA copies assayed by qPCR using the universal 16S primer set that was also used for Illumina amplicon sequencing: Forward primer: 5′-CAACGCGWRGAACCTTACC-3′ and Reverse primer: 5′-CRRCACGAGCTGACGAC-3′.

## Results

### Differences in GI taxonomic profiles by disease status and medication

We characterized the bacterial composition of fecal samples from 30 men (*n* = 6 control, *n* = 3 benign (negative biopsy), *n* = 7 with localized prostate cancer, *n* = 7 with biochemically recurrent prostate cancer, and *n* = 7 with metastatic prostate cancer). Table 1 contains the clinical details of the men included in the study as well as the type of ATT administered at the time of sample collection. Most of the men included in this study were Caucasian (White) and, importantly, there was no statistical difference in patient age or body mass index (BMI) among the medication groups (*p* = 0.4 and *p* = 0.9, respectively, Kruskal–Wallis test). A complete list of all medications and supplements that the men were taking at the time of sample collection is provided in Supplementary Table S1.

**Table 1:** Clinical details of the men included in the study as well as the type of ATT administered at the time of sample collection

	No. of patients	Mean age (range, years)	Mean PSA (range, ng/mL)	Race (no. of patients)^a^
Status				
Control	6	68 (52–80)	5.1 (0.4–20.7)	B (1) W (5)
Benign	3	70 (64–78)	7.3 (4.4–10.1)	B (2) W (1)
Localized cancer	7	60.1 (53–71)	8.4 (3.8–13.5)	B (1) W (6)
Biochemical recurrence	7	64.4 (54–72)	2.4 (0–5.6)	B (1) W (6)
Metastatic hormone-sensitive	2	58 (51–65)	0.25 (0.2–0.3)	W (2)
Metastatic castration-resistant	5	74 (64–85)	8.7 (0–24.5)	W (5)
Medication				Mean BMI (range, kg/m^**2**^)
NoMeds	16	64.1 (52–80)	6.6 (0.4–20.7)	28.05 (19.73–41.80)
GNRH agonist/antagonist	5	64.8 (54–69)	4.6 (0–13.2)	28.66 (22.05–37.67)
Oral ATT	9	69.7 (51–85)	4.9 (0–24.5)	27.25 (21.45–32.07)

*NoMeds* not on ADT, *ATT* androgen axis-targeted therapy

^a^Self-reported as black (B) or white (W). No other races included

The taxonomic profiles of each sample are depicted in Supplemental Figure S1. There was a significant difference in alpha diversity in samples from men with versus without prostate cancer, irrespective of medication status (*p* < 0.05 using multiple statistical models and measures of alpha diversity, Supplementary Table S2), meaning that the total number of species (or species “richness”) and the presence of rare individual species was greater in men without a prostate cancer diagnosis than in men with cancer. No significant differences in alpha diversity by medication status were observed (Supplementary Table S3).

**Fig. 1:**
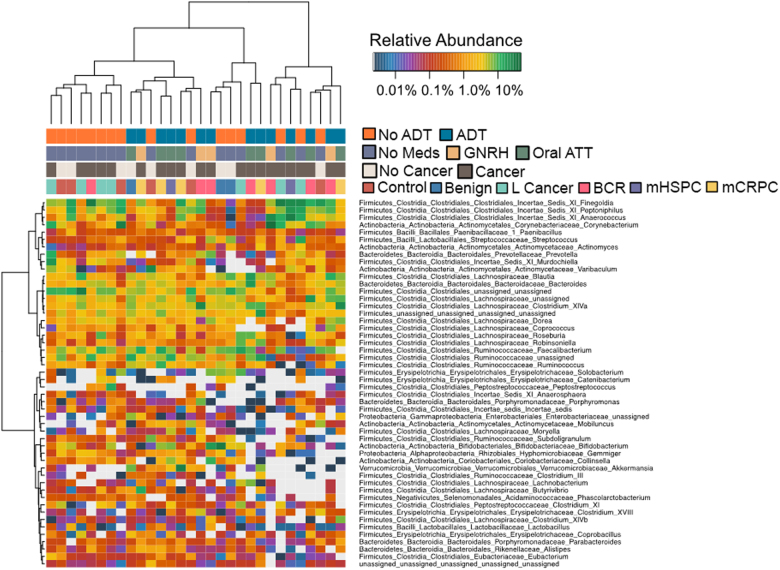
Unsupervised clustering (log-transformed) of 16S rDNA Illumina-sequencing results from fecal samples by genus. The dendrogram was based on hierarchical clustering of the Euclidean distance between samples in the combined groups. L cancer localized prostate cancer, BCR biochemically recurrent prostate cancer, mHSPC metastatic hormone-sensitive prostate cancer, mCRPC metastatic castration-resistant prostate cancer. No cancer = no clinical and/or biopsy proven diagnosis of cancer (control and benign groups)

**Fig. 2:**
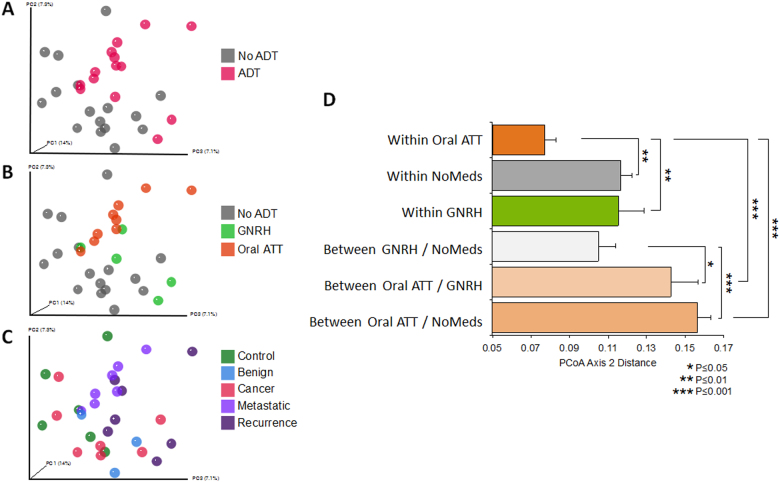
Principal coordinates analysis (PCoA) and beta diversity (unweighted UniFrac) of each fecal sample bacterial profile, analyzed by the indicated groups. **a**–**c** Principal coordinate axis 2 showed the most distinction between medication groups, so statistical comparisons were limited to this dimension. **d** Statistical comparison of beta diversity between the indicated groups (Mann–Whitney test). Shown is the mean unweighted UniFrac distance (+SEM)

Comparison of taxonomic profiles indicated clustering that associated with medication status (ADT versus no ADT), but not with respect to cancer status (no cancer versus cancer, Fig. 1). Of interest, one patient in the recurrence group who had not yet started on ADT at the time of sample collection grouped with the “No ADT” samples (Fig. 1). In PCoA (a measure of relatedness between samples), we observed that samples from men undergoing ADT grouped separately from men who were not undergoing ADT (Fig. 2a). This was particularly pronounced for men taking oral ATT (Fig. 2b). Of interest, men with prostate cancer (localized, biochemically recurrent, or metastatic groups) separated distinctly from men without cancer (control and benign groups) in PCoA (Fig. 2c), a result that was also statistically significant in PERMANOVA (Adonis) model testing (*p* = 0.02). The between-sample distances (beta diversity) were smallest within the oral ATT group compared to the GNRH and NoMeds groups, meaning that the species profiles among the fecal samples within the oral ATT group were most similar to each other, and that a specific taxon or taxa distinguish them from the other groups (Fig. 2d). The greatest beta diversity was observed between the NoMeds group and the oral ATT group (Fig. 2d), meaning that the samples from these two groups were most dissimilar to each other.

### Differentially abundant species in the GI microbiota of men taking oral ATT

Since we observed significant differences in beta diversity by ADT status and type in PCoA, we next determined if particular species of GI microbiota were differentially abundant between medication categories. As shown in Table 2, several species of bacteria were differentially abundant in terms of the proportion of sequencing reads that matched the species/OTU obtained from the samples across different treatment categories. Notably, species, such as *Akkermansia muciniphila*, *Ruminococcaceae* spp., and *Lachnospiraceae* spp., were significantly more abundant in the fecal samples of men taking oral ATT. When analyzed at the bacterial family level, we again observed a significant greater abundance of sequencing reads assigned to the bacterial families *Verrucomicrobiaceae* (of which *Akkermansia muciniphila* is one of the few members), *Lachnospiraceae*, and others in the oral ATT group (Table 2). There was also a significant decrease in the abundance of sequencing reads assigned to bacterial families such as *Brevibacteriaceae*, *Erysipelotrichaceae*, and *Streptococcaceae* in men receiving ADT versus no ADT and specifically in the oral ATT group versus men not undergoing ADT (Table 2).

**Table 2:** Select differentially abundant species or families of GI microbiota in men with or without hormonal therapy

							Negative binomial test (DESeq)					
	Mean values^a^			Standard errors			NoMeds vs GNRH		NoMeds vs oral ATT		GNRH vs oral ATT	
	NoMeds (*n*^b^)	GNRH (*n*)	Oral ATT (*n*)	NoMeds	GNRH	Oral ATT	*P* value	FDR adj *P*	*P* value	FDR adj *P*	*P* value	FDR adj *P*
Species/OTUs												
Akkermansia muciniphila	0.002 (8)	0.003 (3)	0.055 (6)	0.001	0.002	0.034	0.623	0.797	0.002	0.012	0.048	0.173
Ruminococcaceae_unassigned	0.011 (16)	0.001 (4)	0.030 (9)	0.004	0.001	0.012	0.051	0.181	0.049	0.179	0.010	0.051
Blautia wexlerae	0.012 (16)	0.029 (5)	0.026 (9)	0.002	0.019	0.017	0.030	0.121	0.023	0.098	0.907	0.929
Ruminococcaceae_unassigned	0.005 (16)	0.003 (4)	0.018 (9)	0.002	0.001	0.007	0.400	0.690	0.027	0.115	<0.001	0.002
otu0:Clostridium oroticum	0.003 (14)	0.048 (5)	0.017 (9)	0.001	0.044	0.007	0.000	<0.001	0.001	0.007	0.145	0.373
Lachnospiraceae_Clostridium_XlVa	0.006 (14)	0.008 (4)	0.016 (8)	0.001	0.004	0.011	0.515	0.713	0.032	0.129	0.358	0.645
Clostridiales_unassigned	0.056 (14)	0.032 (5)	0.016 (8)	0.017	0.015	0.005	0.401	0.690	0.010	0.052	0.219	0.490
otu3527:Robinsoniella peoriensis	0.001 (15)	<0.001 (4)	0.016 (8)	<0.001	<0.001	0.008	0.113	0.328	<0.001	<0.001	<0.001	<0.001
Anaerococcus tetradius	0.001 (10)	0.006 (3)	0.010 (3)	<0.001	0.006	0.008	0.045	0.163	0.006	0.032	0.769	0.832
Bacteroides stercoris	0.001 (11)	<0.001 (3)	0.009 (2)	0.001	<0.001	0.007	0.507	0.707	0.008	0.042	0.084	0.263
Family												
Brevibacteriaceae	0.002	<0.001	<0.001	0.002	<0.001	<0.001	0.076	0.240	<0.001	<0.001	<0.001	<0.001
Clostridiales_Incertae_Sedis_XIII	<0.001	<0.001	0.001	<0.001	<0.001	0.001	0.050	0.222	<0.001	<0.001	0.245	0.517
Staphylococcaceae	<0.001	0.001	0.003	<0.001	0.001	0.002	0.040	0.218	<0.001	<0.001	0.382	0.613
Clostridiales_unassigned	0.087	0.050	0.029	0.020	0.017	0.007	0.217	0.415	0.001	0.008	0.158	0.405
Verrucomicrobiaceae	0.002	0.003	0.056	0.001	0.002	0.034	0.632	0.766	0.002	0.015	0.048	0.160
Oxalobacteraceae	<0.001	<0.001	<0.001	<0.001	<0.001	<0.001	0.472	0.693	0.004	0.030	0.183	0.455
Bacillales_unassigned	<0.001	<0.001	0.001	<0.001	<0.001	0.001	0.507	0.701	0.005	0.030	0.022	0.105
Aerococcaceae	0.001	0.004	0.004	<0.001	0.002	0.002	0.017	0.107	0.005	0.032	0.872	0.895
Propionibacteriaceae	<0.001	0.001	<0.001	<0.001	0.001	<0.001	0.155	0.368	0.014	0.065	0.002	0.020
Erysipelotrichaceae	0.039	0.028	0.017	0.009	0.015	0.004	0.675	0.766	0.016	0.071	0.272	0.523
Selenomonadales_unassigned	<0.001	0.003	0.001	<0.001	0.003	0.001	0.001	0.014	0.020	0.084	0.324	0.554
Streptococcaceae	0.021	0.014	0.007	0.010	0.012	0.002	0.817	0.872	0.025	0.094	0.260	0.517
Lachnospiraceae	0.176	0.272	0.305	0.027	0.050	0.064	0.046	0.220	0.027	0.097	0.650	0.806
Prevotellaceae	0.020	0.034	0.006	0.008	0.016	0.002	0.328	0.553	0.037	0.118	0.005	0.044

*NoMeds* not on ADT, *GNRH* only on GNRH agonist/antagonist, *Oral* *ATT* taking an oral androgen axis-targeted therapy, *FDR adj P* false discovery rate adjusted P value

^a^Mean relative abundance

^b^Number of patients the species/OTU or bacterial family was identified in

**Fig. 3:**
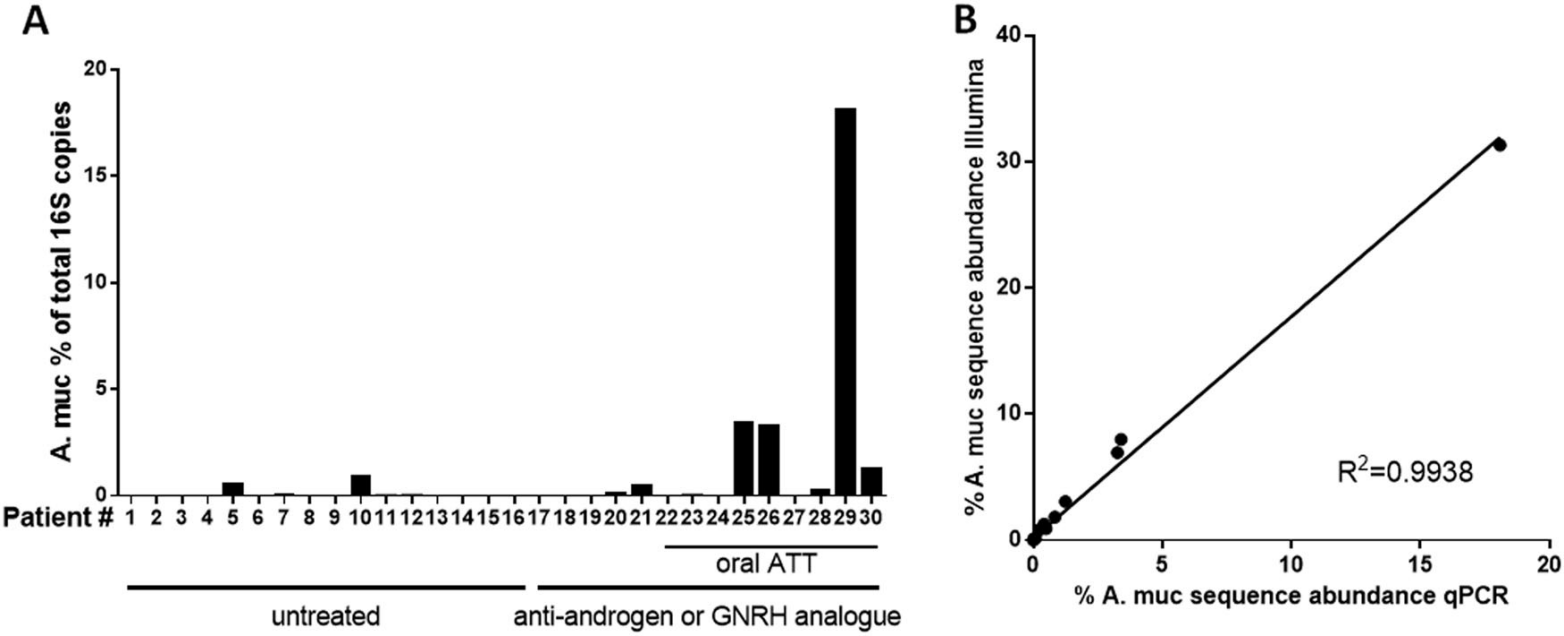
Quantitative PCR (qPCR) for *Akkermansia muciniphila*. **a** Confirmation of enriched abundance of this species in the GI microbiota of men taking oral ATT versus the other men included in the study. **b** The qPCR results were in strong correlation (*R*^2^ = 0.9938) with the results obtained by 16S rDNA Illumina amplicon sequencing

As *Akkermansia muciniphila* is a species of particular recent interest with respect to treatment response to anti-PD-1 immunotherapy in patients with epithelial tumors, we verified the differential abundance of *A. muciniphila* in men taking oral ATT using an independent qPCR assay. These analyses confirmed that *A. muciniphila* was significantly more prevalent in the men who were taking oral ATT. Moreover, the results showed high correlation with our Illumina amplicon sequencing results (Fig. 3).

### Enrichment of GI microbiota predicted to contain genes related to steroid/hormone biosynthesis in men taking oral ATT

We next performed functional inference analyses using PICRUSt [20]. Functional pathways involving steroid/hormone biosynthesis were significantly enriched within the oral ATT group compared to the no ADT (NoMeds) group (Table 3). Other pathways of interest that were more prevalent in the oral ATT group versus the no ADT group included caffeine metabolism and glycosaminoglycan degradation (Table 3).

**Table 3:** Functional inference of microbial gene content in fecal specimens

	Mean values (relative abundance)				NoMeds vs oral ATT
KEGG level 3—Categories enriched in oral ATT vs NoMeds	NoMeds (*n* = 16)	GNRH (*n* = 5)	Oral ATT (*n* = 9)	Ratio oral ATT to NoMeds	*P* value (npdiff)
Metabolism;Lipid_Metabolism; Steroid_biosynthesis	5.06E−06	6.04E−06	9.49E−05	18.75	0.0044
Metabolism;Lipid_Metabolism; Steroid_hormone_biosynthesis	3.67E−05	4.15E−05	1.91E−04	5.2	0.0012
Metabolism;Biosynthesis_of_Other_Secondary_Metabolites; Caffeine_metabolism	3.44E−06	1.21E−06	3.09E−05	8.98	0.0188
Metabolism;Xenobiotics_Biodegradation_and_Metabolism; Fluorobenzoate_degradation	8.65E−06	4.65E−06	6.48E−05	7.5	0.0194
Metabolism;Glycan_Biosynthesis_and_Metabolism; Glycosaminoglycan_degradation	2.13E−04	3.02E−04	4.98E−04	2.34	0.0022
Metabolism;Xenobiotics_Biodegradation_and_Metabolism; Atrazine_degradation	1.93E−04	2.06E−04	3.64E−04	1.88	0.0012
Metabolism;Glycan_Biosynthesis_and_Metabolism; Glycosphingolipid_biosynthesis_-_ganglio_series	1.14E−04	1.79E−04	2.13E−04	1.88	0.0212
Metabolism;Biosynthesis_of_Other_Secondary_Metabolites;Flavonoid_biosynthesis	5.55E−05	8.19E−05	9.93E−05	1.79	0.0218
Metabolism;Biosynthesis_of_Other_Secondary_Metabolites;Penicillin_and_cephalosporin_biosynthesis	1.34E−04	1.53E−04	2.27E−04	1.7	0.0068
Cellular_Processes;Transport_and_Catabolism;Lysosome	4.69E−04	5.08E−04	8.03E−04	1.71	0.0048
Metabolism;Biosynthesis_of_Other_Secondary_Metabolites;beta-Lactam_resistance	2.17E−04	2.63E−04	2.93E−04	1.35	0.029
Unclassified;Cellular_Processes_and_Signaling; Cell_motility_and_secretion	1.25E−03	1.32E−03	1.65E−03	1.32	0.0026
Metabolism;Glycan_Biosynthesis_and_Metabolism; Glycosphingolipid_biosynthesis_-_globo_series	6.69E−04	7.72E−04	8.58E−04	1.28	0.0278
Unclassified;Cellular_Processes_and_Signaling; Inorganic_ion_transport_and_metabolism	1.59E−03	1.92E−03	1.98E−03	1.25	0.0052

*NoMeds* not on ADT, *GNRH* only on GNRH agonist/antagonist, *Oral* *ATT* taking an oral androgen axis-targeted therapy, *npdiff* nonparametric difference test

## Discussion

Our study aimed to assess the compositional profile of the GI microbiota in men with and without a diagnosis of prostate cancer and with and without treatment with ATT. We report initial evidence that the alpha diversity of the GI microbiota is greater in men without a prostate cancer diagnosis, and that there were measurable differences in the bacterial composition of the GI microbiota in men undergoing treatment with ATT.

### Cancer therapies and GI microbiota

The ability of cancer therapies to affect and change the composition of the GI microbiota is not well studied. Interestingly, a screen of more than 1000 marketed non-antibiotic drugs against 40 representative GI bacterial strains found that nearly a quarter inhibited bacterial growth [21]. Chemotherapy and immunotherapy have been shown to induce dysbiosis (a pathogenic microbial imbalance) of the GI microbiota in rodent models [7, 22]. The most mature data involving longitudinal studies are in patients with non-Hodgkin’s lymphoma undergoing bone marrow transplant conditioning chemotherapy, which induced pathogenic shifts in the GI microbiota that were associated with treatment toxicities [23, 24]. A study in pediatric acute myeloid leukemia patients receiving chemotherapy demonstrated direct bacteriostatic effect of chemotherapeutics, as well as outgrowth of pathogenic enterococci that could not be fully explained by concurrent use of antibiotics [25]. Compositional changes to the GI microbiota induced by chemotherapy or immunotherapy could conceivably impact factors such as the local inflammatory environment in the intestinal tract, systemic inflammatory effects, and/or the efficacy of any subsequently administered cancer therapies.

### The microbiome and systemic hormone levels

It has been reported that steroid biosynthesis occurs in prokaryotes [26, 27], and that certain species of bacteria are capable of metabolizing estrogen and androgen precursors and catabolizing estrogens and androgens thereby affecting systemic levels of these hormones [28–30]. Altering the gastrointestinal flora in a mouse model of type 1 diabetes impacted testosterone levels, as well as the development of type 1 diabetes [12]. In another study, mice consuming a diet rich in the commonly used probiotic strain *Lactobacillus reuteri* had a reduced systemic inflammatory state through reduction of IL-17, and an increase in serum testosterone levels [31]. On the converse, the microbiome can also be affected by hormone levels, as another mouse study showed that castrating mice induced alterations in GI microbiota composition, and subsequent development of abdominal obesity [16]. Intriguingly, this study by Harada et al. implies that the GI microbiota may mediate several of the side effects associated with ADT, including obesity and the metabolic syndrome. In our study, we found that men taking oral ATT had a different GI microbiota composition than men taking GNRH agonists/antagonists alone or men not undergoing ADT. Functional pathway inference of the species present in the fecal microbiota of men taking oral ATT indicated an intriguing possibility that the species capable of steroid/hormone biosynthesis are more abundant in the GI flora when men are taking these oral medications (Table 3). This finding, if confirmed, could have important implications and perhaps represent a mechanism for potential alternative pathways for production of steroid metabolites that could influence treatment response to oral ATT. Critical follow-up studies will correlate the presence of GI bacterial species capable of steroid/hormone biosynthesis to circulating hormone levels.

### Oral ATT, GI microbiota, and immunotherapy

Much excitement has been generated after the publication of a series of human studies in melanoma patients [9, 11] and in patients with epithelial tumors [10], all indicating that the presence of certain types of bacteria including *Ruminococcaceae, Bifidobacteriaceae*, and *Akkermansia muciniphila* are associated with a positive response to anti-PD-1 immunotherapy. In our study, we observed overrepresentation of these same species (*Ruminococcaceae* and particularly *Akkermansia muciniphila*) in the fecal microbiota of men taking oral ATT (Table 2). Although preliminary, we speculate that our results might represent one potential explanation for the report of responses to anti-PD-1 immunotherapy observed in men with metastatic prostate cancer who have progressed on enzalutamide [32].

There are several limitations to our study including the relatively small sample size and the lack of longitudinal sampling. Important follow-up studies will include samples collected prior to start of therapy and then longitudinally after therapy initiation. Such studies will further strengthen our hypothesis that ATT is responsible for the compositional differences that we observed, as opposed to other factors that can influence the composition of the GI microbiome such as diet or stress levels. Furthermore, we observed a significant decrease in GI microbiota alpha diversity in patients with prostate cancer that was independent of medication status. Decreased diversity in GI microbiota has been reported as a risk factor for several other types of disease as well as “Western” lifestyle [33]. Our results should be taken with caution, however, as many of the men with prostate cancer in this study had undergone prior treatments that could have conceivably influenced the diversity of the GI microbiota. Our results prompt further examination of GI microbiota diversity as a risk factor for prostate cancer in larger patient cohorts.

In conclusion, our study provides preliminary evidence that the GI microbiota may be different in men undergoing treatment with androgen receptor axis-targeted therapies commonly used to treat prostate cancer. We hypothesize that these compositional differences may influence treatment response to oral ATT or to subsequent treatments such as immunotherapy. Future longitudinal studies pre-, during, and post-therapy are warranted to confirm the degree to which the GI microbiota are altered and to assess whether these alterations are correlated to prostate cancer treatment responses. Collectively, these studies could determine whether the GI microbiome is both essential for therapeutic efficacy and whether it could serve as a target that could be modulated to enhance treatment response.

## Electronic supplementary material

Supplemental legends

Supplemental Methods

Supplementary Table S1

Supplementary Table S2

Supplementary Table S3

Supplemental Figure S1
