# Supplementary material for: Compositional differences in gastrointestinal microbiota in prostate cancer patients treated with androgen axis-targeted therapies
Source: Prostate Cancer Prostatic Dis. 2018 Jul 9;21(4):539–48. doi: 10.1038/s41391-018-0061-x (PMC6283851; doi:10.1038/s41391-018-0061-x)
Supplement: Supplementary file 1 — Supplemental legends [file 41391_2018_61_MOESM1_ESM.docx]

Supplemental Figure S1. Bacterial families identified in fecal samples from the 30 men in the study organized by their state of prostate cancer (left) or by their current medication status (right). Stacked bar plots represent the sequence abundances of the 15 most abundant family-level taxa identified in the fecal samples. Percent sequence abundances given as the number of reads matching a given bacterial family per total reads for that sample.
