# Supplementary material for: Compositional differences in gastrointestinal microbiota in prostate cancer patients treated with androgen axis-targeted therapies
Source: Prostate Cancer Prostatic Dis. 2018 Jul 9;21(4):539–48. doi: 10.1038/s41391-018-0061-x (PMC6283851; doi:10.1038/s41391-018-0061-x)
Supplement: Supplementary file 2 — Supplemental Methods [file 41391_2018_61_MOESM2_ESM.docx]

*Rectal swab collection protocol for microbiome analysis*.

**Reagents and Supplies Needed:**

Regular flocked swab, plastic applicator, sterile in dry tube (Copan, catalog #552C), 2- 3mL cryovials

**Protocol:**

1. Remove swab from plastic tube. Hold the swab by the end of the plastic handle and take care not to touch the cotton tip of the swab to any surfaces or with fingers.
2. Insert the swab directly into the rectum and approximately 3 cm into the anal canal. Rotate the swab and withdraw. **If fecal matter is on the swab, it has been done correctly.**
3. Place the swab back into the plastic tube.

**Storage:**

1. Break swabs at neck and store in 3 mL cryovials at -80 degrees Celsius.

*16S rRNA gene library generation and HiSeq sequencing*. The V6 hypervariable region of the 16S rRNA gene was amplified using a two-step PCR strategy as previously described [1]. PCR products were visualized on an agarose gel, gel extracted, and pooled before submitting to the SKCCC Next Generation Sequencing Core at Johns Hopkins for next generation sequencing on an Illumina HiSeq instrument.

*Sequence analysis*. Raw paired-end reads were merged into consensus sequences using FLASH requiring a minimum 20 bp overlap and a 5% maximum mismatch density, and subsequently filtered for quality (targeting an error rate < 0.1%) and length (minimum 60 bp) using Trimmomatic and QIIME [2]. Passing sequences were then trimmed of primers, evaluated for chimeras with UCLUST [3] (*de novo* mode), and filtered for host-associated contaminant using Bowtie2 [4] searches of NCBI Homo sapiens Annotation Release 106. Additionally chloroplast and mitochondrial contaminants were detected and filtered using the RDP classifier with a confidence threshold of 50%. Sequencing contaminants as determined by negative controls were removed from the sample data set as previously described [1]. High-quality clean 16S rDNA sequences were then subjected to high-resolution taxonomic assessment using Resphera Insight [5-7]. Resphera Insight attempts to achieve species-level resolution when possible, however when the underlying statistical model indicates divergence from all known species, the algorithm will perform *de novo* clustering into operational taxonomic units (OTUs) follow by taxonomic assignment of the OTU representatives; these non-species assignments are denoted with an “otu” label and include the closest related species identified. Functional inference was performed using PICRUSt [8]. PICRUSt is a bioinformatics software package designed to predict metagenome functional content from 16S rDNA surveys such as the one performed in this study.

**References**

1. Shrestha E, White JR, Yu S-H, Kulac I, Ertunc O, De Marzo AM *et al*. Profiling the urinary microbiome in men with positive versus negative biopsies for prostate cancer. *The Journal of Urology* 2018; **199**(1)**:** 161-171.

2. Caporaso JG, Kuczynski J, Stombaugh J, Bittinger K, Bushman FD, Costello EK *et al*. QIIME allows analysis of high-throughput community sequencing data. *Nat Methods* 2010; **7**(5)**:** 335-336.

3. Edgar RC. Search and clustering orders of magnitude faster than BLAST. *Bioinformatics* 2010; **26**(19)**:** 2460-2461.

4. Langmead B, Salzberg SL. Fast gapped-read alignment with Bowtie 2. *Nat Methods* 2012; **9**(4)**:** 357-359.

5. Abernethy MG, Rosenfeld A, White JR, Mueller MG, Lewicky-Gaupp C, Kenton K. Urinary microbiome and cytokine levels in women with interstitial cystitis. *Obstetrics and gynecology* 2017; **129**(3)**:** 500-506.

6. Ottesen A, Ramachandran P, Reed E, White JR, Hasan N, Subramanian P *et al*. Enrichment dynamics of Listeria monocytogenes and the associated microbiome from naturally contaminated ice cream linked to a listeriosis outbreak. *BMC Microbiol* 2016; **16**(1)**:** 275.

7. Daquigan N, Grim CJ, White JR, Hanes DE, Jarvis KG. Early recovery of Salmonella from food using a 6-hour non-selective pre-enrichment and reformulation of tetrathionate broth. *Frontiers in Microbiology* 2016; **7:** 2103.

8. Langille MGI, Zaneveld J, Caporaso JG, McDonald D, Knights D, Reyes JA *et al*. Predictive functional profiling of microbial communities using 16S rRNA marker gene sequences. *Nature Biotechnology* 2013; **31:** 814.
