# Supplementary material for: Compositional differences in gastrointestinal microbiota in prostate cancer patients treated with androgen axis-targeted therapies
Source: Prostate Cancer Prostatic Dis. 2018 Jul 9;21(4):539–48. doi: 10.1038/s41391-018-0061-x (PMC6283851; doi:10.1038/s41391-018-0061-x)
Supplement: Supplementary file 3 — Supplementary Table S1 [file 41391_2018_61_MOESM3_ESM.docx]

**Supplementary Table S1**. List of all GU medications and supplements that the men were taking at time of sample collection.

| **Patient #** | **ADT Medication Category** | **Drug Name** | **Medication duration (months)** |
| --- | --- | --- | --- |
| **1** | Untreated | None |  |
| **2** | Untreated | None |  |
| **3** | Untreated | Prednisone | 3 |
| **4** | Untreated | Flomax/Tamsulosin | 10 |
| **5** | Untreated | Saw palmetto | Unknown |
| **6** | Untreated | None |  |
| **7** | Untreated | Flomax/Tamsulosin | Unknown |
| **8** | Untreated | Saw palmetto | Unknown |
| **9** | Untreated | None |  |
| **10** | Untreated | Uroxatral/Alfuzosin | Unknown |
| **11** | Untreated | None |  |
| **12** | Untreated | None |  |
| **13** | Untreated | None |  |
| **14** | Untreated | None |  |
| **15** | Untreated | None |  |
| **16** | Untreated | Cardura/doxazosin | Unknown |
| **17** | GNRH | Zoladex | Unknown |
| **18** | GNRH | Zoladex | 3 |
| **19** | GNRH | Zoladex | 15 |
| **20** | GNRH | Trelstar/triptorelin | 48 |
| **21** | GNRH | Trelstar/triptorelin | 6 |
| **22** | Oral AA | Casodex/Bicalutamide | Unknown |
|  |  | Flomax/Tamsulosin | 19 |
|  |  | Lupron | 18 |
|  |  | Trelstar/triptorelin | 18 |
| **23** | Oral AA | Casodex/Bicalutamide | 1 |
| **24** | Oral AA | Abiraterone/zytiga | 6 |
|  |  | Degarelix/Firmagon | 6 |
|  |  | Prednisone | 6 |
| **25** | Oral AA | Proscar/finasteride | Unknown |
|  |  | Flomax/Tamsulosin | Unknown |
| **26** | Oral AA | Abiraterone/zytiga | 3 |
|  |  | Zoladex | Unknown |
| **27** | Oral AA | Abiraterone/zytiga | 2 |
|  |  | Lupron | 34 |
|  |  | Enzalutamide/xtandi | 2 |
|  |  | Prednisone | 2 |
| **28** | Oral AA | ARN-509 | Unknown |
|  |  | Trelstar/triptorelin | Unknown |
|  |  | Flomax/Tamsulosin | Unknown |
| **29** | Oral AA | ARN-509 | Unknown |
|  |  | Eligard | Unknown |
| **30** | Oral AA | Casodex/Bicalutamide | 3 |
|  |  | Palbociclib/Ibrance | 3 |
|  |  | Zoladex | 3 |
