# Supplementary material for: Compositional differences in gastrointestinal microbiota in prostate cancer patients treated with androgen axis-targeted therapies
Source: Prostate Cancer Prostatic Dis. 2018 Jul 9;21(4):539–48. doi: 10.1038/s41391-018-0061-x (PMC6283851; doi:10.1038/s41391-018-0061-x)
Supplement: Supplementary file 4 — Supplementary Table S2 [file 41391_2018_61_MOESM4_ESM.docx]

**Supplementary Table S2**. Alpha diversity measures compared between No cancer and Cancer groups. Statistical results include three response variable family distributions (1. normal gaussian, 2. log-normal gaussian and 3. gamma identity-link).

|  | **MEAN VALUES** | | **GLM Results (Gaussian family assumption)** | | |
| --- | --- | --- | --- | --- | --- |
| **Alpha Diversity Measure** | **No Cancer (n=9)** | **Cancer (n=21)** | **Feature** | **(Intercept)** | **pvalue(fdradj pvalue, ERC*)** |
| chao1 | 775.250 | 517.496 | chao1 | 2.598e-11(3.118e-11,775.250) | **0.006(0.039,-257.754)** |
| goods_coverage | 0.996 | 0.997 | goods_coverage | 1.265e-75(1.518e-74,0.996) | **0.008(0.0392,0.001)** |
| PD_whole_tree | 109.193 | 88.488 | PD_whole_tree | 6.590e-16(1.130e-15,109.193) | **0.015(0.039,-20.706)** |
| fisher_alpha | 81.468 | 56.354 | fisher_alpha | 2.242e-10(2.445e-10,81.468) | **0.019(0.039,-25.114)** |
| observed_species | 507.444 | 371.333 | observed_species | 1.058e-11(1.476e-11,507.444) | **0.020(0.039,-136.111)** |
| margalef | 47.367 | 34.637 | margalef | 1.107e-11(1.476e-11,47.367) | **0.020(0.039,-12.730)** |
| gini_index | 0.984 | 0.988 | gini_index | 6.636e-58(3.981e-57,0.984) | 0.124(0.213,0.003) |
| shannon | 5.523 | 5.168 | shannon | 1.040e-20(2.080e-20,5.523) | 0.189(0.257,-0.355) |
| brillouin_d | 3.807 | 3.566 | brillouin_d | 9.258e-21(2.080e-20,3.807) | 0.192(0.257,-0.242) |
| simpson_reciprocal | 22.587 | 17.956 | simpson_reciprocal | 6.872e-07(6.872e-07,22.587) | 0.284(0.332,-4.632) |
| strong | 0.784 | 0.774 | strong | 2.866e-37(1.1462e-36,0.784) | 0.305(0.332,-0.010) |
| mcintosh_d | 0.767 | 0.742 | mcintosh_d | 8.340e-24(2.502e-23,0.767) | 0.382(0.382,-0.025) |
|  |  |  | **GLM Results (log-normal family assumption)** | | |
|  |  |  | **Feature** | **(Intercept)** | **pvalue(fdradj pvalue, ERC)** |
|  |  |  | chao1 | 4.407e-33(2.644e-32,6.653) | **0.005(0.032,-0.404)** |
|  |  |  | goods_coverage | 5.086e-10(6.103e-10,-0.004) | **0.008(0.032,0.001)** |
|  |  |  | PD_whole_tree | 3.721e-34(4.466e-33,4.693) | **0.012(0.032,-0.210)** |
|  |  |  | fisher_alpha | 6.250e-27(1.250e-26,4.400) | **0.0156(0.032,-0.369)** |
|  |  |  | observed_species | 9.178e-33(3.671e-32,6.229) | **0.0159(0.032,-0.312)** |
|  |  |  | margalef | 5.722e-27(1.250e-26,3.858) | **0.0159(0.0318,-0.313)** |
|  |  |  | gini_index | 2.106e-09(2.234e-09,-0.0159) | 0.125(0.214,0.003) |
|  |  |  | shannon | 4.658e-27(1.250e-26,1.709) | 0.183(0.250,-0.066) |
|  |  |  | brillouin_d | 3.520e-24(6.035e-24,1.337) | 0.187(0.250,-0.066) |
|  |  |  | simpson_reciprocal | 4.969e-18(6.625e-18,3.117) | 0.269(0.323,-0.229) |
|  |  |  | strong | 2.375e-20(3.562e-20,-0.244) | 0.303(0.331,-0.013) |
|  |  |  | mcintosh_d | 2.234e-09(2.234e-09,-0.265) | 0.379(0.379,-0.033) |
|  |  |  | **GLM Results (Gamma family assumption)** | | |
|  |  |  | **Feature** | **(Intercept)** | **pvalue(fdradj pvalue, ERC)** |
|  |  |  | chao1 | 6.792e-09(8.151e-09,775.250) | **0.019(0.085,-257.754)** |
|  |  |  | goods_coverage | 1.238e-75(1.485e-74,0.996) | **0.008(0.085,0.001)** |
|  |  |  | PD_whole_tree | 3.951e-14(6.774e-14,109.193) | **0.0268(0.085,-20.706)** |
|  |  |  | fisher_alpha | 3.056e-08(3.334e-08,81.468) | **0.0426(0.0852,-25.114)** |
|  |  |  | observed_species | 1.011e-09(1.421e-09,507.444) | **0.0387(0.0852,-136.111)** |
|  |  |  | margalef | 1.066e-09(1.421e-09,47.367) | **0.039(0.0852,-12.730)** |
|  |  |  | gini_index | 6.226e-58(3.735e-57,0.984) | 0.124(0.212,0.003) |
|  |  |  | shannon | 3.946e-20(7.893e-20,5.523) | 0.202(0.274,-0.355) |
|  |  |  | brillouin_d | 3.456e-20(7.893e-20,3.807) | 0.206(0.274,-0.242) |
|  |  |  | simpson_reciprocal | 6.551e-06(6.551e-06,22.587) | 0.323(0.353,-4.632) |
|  |  |  | strong | 3.768e-37(1.507e-36,0.784) | 0.308(0.353,-0.010) |
|  |  |  | mcintosh_d | 1.622e-23(4.866e-23,0.767) | 0.389(0.389,-0.025) |

*ERC= estimated regression coefficient
