# Supplementary material for: Compositional differences in gastrointestinal microbiota in prostate cancer patients treated with androgen axis-targeted therapies
Source: Prostate Cancer Prostatic Dis. 2018 Jul 9;21(4):539–48. doi: 10.1038/s41391-018-0061-x (PMC6283851; doi:10.1038/s41391-018-0061-x)
Supplement: Supplementary file 5 — Supplementary Table S3 [file 41391_2018_61_MOESM5_ESM.docx]

**Supplementary Table S3**. Alpha diversity measures compared between medication groups. Statistical results include three response variable family distributions (1. normal gaussian, 2. log-normal gaussian and 3. gamma identity-link).

|  | **MEAN VALUES** | | | **GLM Results (Gaussian family assumption)** | | | |
| --- | --- | --- | --- | --- | --- | --- | --- |
| **Alpha Diversity Measure** | **NoMeds** | **GNRH** | **Oral ATT** | **Feature** | **(Intercept)** | **NoMeds v GNRH, *** | **NoMeds v Oral ATT, *** |
| PD_whole_tree | 98.886 | 84.072 | 93.161 | PD_whole_tree | 1.354e-16(2.321e-16,98.886) | 0.198(0.688,-14.814) | 0.536(0.724,-5.725) |
| goods_coverage | 0.997 | 0.998 | 0.997 | goods_coverage | 2.577e-75(3.093e-74,0.997) | 0.227(0.688,0.001) | 0.280(0.724,0.001) |
| simpson_reciprocal | 18.225 | 15.662 | 23.382 | simpson_reciprocal | 2.431e-07(2.431e-07,18.225) | 0.643(0.701,-2.563) | 0.256(0.724,5.157) |
| chao1 | 648.468 | 501.882 | 551.085 | chao1 | 5.320e-11(6.384e-11,648.468) | 0.259(0.688,-146.586) | 0.354(0.724,-97.383) |
| mcintosh_d | 0.740 | 0.735 | 0.774 | mcintosh_d | 4.5166e-26(1.355e-25,0.740) | 0.878(0.878,-0.006) | 0.260(0.724,0.034) |
| observed_species | 435.625 | 354.800 | 402.333 | observed_species | 7.023e-12(9.862e-12,435.625) | 0.309(0.688,-80.825) | 0.603(0.724,-33.292) |
| margalef | 40.650 | 33.090 | 37.536 | margalef | 7.396e-12(9.862e-12,40.650) | 0.309(0.688,-7.559) | 0.603(0.724,-3.114) |
| fisher_alpha | 67.972 | 54.187 | 62.018 | fisher_alpha | 2.800e-10(3.055e-10,67.972) | 0.3463(0.688,-13.785) | 0.615(0.7241,-5.954) |
| strong | 0.781 | 0.772 | 0.773 | strong | 2.968e-39(1.1873e-38,0.781) | 0.490(0.688,-0.009) | 0.461(0.724,-0.008) |
| shannon | 5.273 | 5.056 | 5.398 | shannon | 1.460e-22(2.921e-22,5.273) | 0.542(0.688,-0.217) | 0.664(0.724,0.126) |
| brillouin_d | 3.636 | 3.489 | 3.724 | brillouin_d | 1.267e-22(2.921e-22,3.636) | 0.545(0.688,-0.148) | 0.658(0.724,0.088) |
| gini_index | 0.986 | 0.988 | 0.986 | gini_index | 7.282e-59(4.369e-58,0.986) | 0.574(0.688,0.002) | 0.937(0.937,-0.0002) |
|  |  |  |  | **GLM Results (log-normal family assumption)** | | | |
|  |  |  |  | **Feature** | **(Intercept)** | **NoMeds v GNRH, *** | **NoMeds v Oral ATT, *** |
|  |  |  |  | PD_whole_tree | 4.801e-34(5.761e-33,4.594) | 0.219(0.688,-0.162) | 0.540(0.723,-0.060) |
|  |  |  |  | goods_coverage | 7.61e-10(7.606e-10,-0.003) | 0.227(0.688,0.0009) | 0.280(0.723,0.0007) |
|  |  |  |  | simpson_reciprocal | 1.254e-17(1.672e-17,2.903) | 0.657(0.717,-0.152) | 0.248(0.723,0.249) |
|  |  |  |  | chao1 | 1.091e-31(4.366e-31,6.475) | 0.296(0.688,-0.256) | 0.368(0.723,-0.163) |
|  |  |  |  | mcintosh_d | 8.816e-13(1.058e-12,-0.300) | 0.878(0.878,-0.008) | 0.257(0.723,0.045) |
|  |  |  |  | observed_species | 5.176e-32(3.106e-31,6.077) | 0.338(0.688,-0.205) | 0.608(0.723,-0.080) |
|  |  |  |  | margalef | 3.089e-26(7.414e-26,3.705) | 0.338(0.688,-0.206) | 0.608(0.723,-0.080) |
|  |  |  |  | fisher_alpha | 8.084e-26(1.617e-25,4.219) | 0.379(0.688,-0.227) | 0.620(0.723,-0.092) |
|  |  |  |  | strong | 5.0325e-23(7.549e-23,-0.248) | 0.491(0.688,-0.011) | 0.462(0.723,-0.010) |
|  |  |  |  | shannon | 2.015e-28(6.046e-28,1.662) | 0.546(0.688,-0.042) | 0.663(0.723,0.0235) |
|  |  |  |  | brillouin_d | 1.481e-25(2.539e-25,1.291) | 0.549(0.688,-0.041) | 0.657(0.723,0.024) |
|  |  |  |  | gini_index | 4.846e-10(5.286e-10,-0.014) | 0.5736(0.688,0.002) | 0.937(0.937,-0.0002) |
|  |  |  |  | **GLM Results (Gamma family assumption)** | | | |
|  |  |  |  | **Feature** | **(Intercept)** | **NoMeds v GNRH, *** | **NoMeds v Oral ATT, *** |
|  |  |  |  | PD_whole_tree | 7.002e-16(1.200e-15,98.886) | 0.176(0.622,-14.814) | 0.547(0.730,-5.725) |
|  |  |  |  | goods_coverage | 2.553e-75(3.064e-74,0.997) | 0.227(0.622,0.0009) | 0.280(0.730,0.0007) |
|  |  |  |  | simpson_reciprocal | 8.444e-08(8.444e-08,18.225) | 0.583(0.636,-2.563) | 0.310(0.730,5.157) |
|  |  |  |  | chao1 | 4.835e-10(5.810e-10,648.468 | 0.222(0.622,-146.586) | 0.357(0.730,-97.383) |
|  |  |  |  | mcintosh_d | 3.266e-26(9.797e-26,0.740) | 0.876(0.876,-0.006) | 0.268(0.730,0.034) |
|  |  |  |  | observed_species | 4.114e-11(5.797e-11,435.625) | 0.276(0.621,-80.825) | 0.614(0.730,-33.292) |
|  |  |  |  | margalef | 4.348e-11(5.797e-11,40.650) | 0.276(0.622,-7.559) | 0.614(0.730,-3.11) |
|  |  |  |  | fisher_alpha | 1.737e-09(1.895e-09,67.972) | 0.311(0.622,-13.785) | 0.626(0.730,-5.954) |
|  |  |  |  | strong | 3.398e-39(1.359e-38,0.781) | 0.488(0.636,-0.009) | 0.460(0.730,-0.008) |
|  |  |  |  | shannon | 1.492e-22(2.984e-22,5.273) | 0.529(0.636,-0.217) | 0.669(0.730,0.126) |
|  |  |  |  | brillouin_d | 1.286e-22(2.984e-22,3.636) | 0.533(0.636,-0.148) | 0.663(0.730,0.088) |
|  |  |  |  | gini_index | 7.216e-59(4.329e-58,0.986) | 0.574(0.636,0.002) | 0.937(0.937,-0.0002) |
| *pvalue(fdradj pvalue, estimated regression coefficient) | | |  |  |  |  |  |
