# Supplementary figures and images for: Compositional differences in gastrointestinal microbiota in prostate cancer patients treated with androgen axis-targeted therapies
Source: Prostate Cancer Prostatic Dis. 2018 Jul 9;21(4):539–48. doi: 10.1038/s41391-018-0061-x (PMC6283851; doi:10.1038/s41391-018-0061-x)

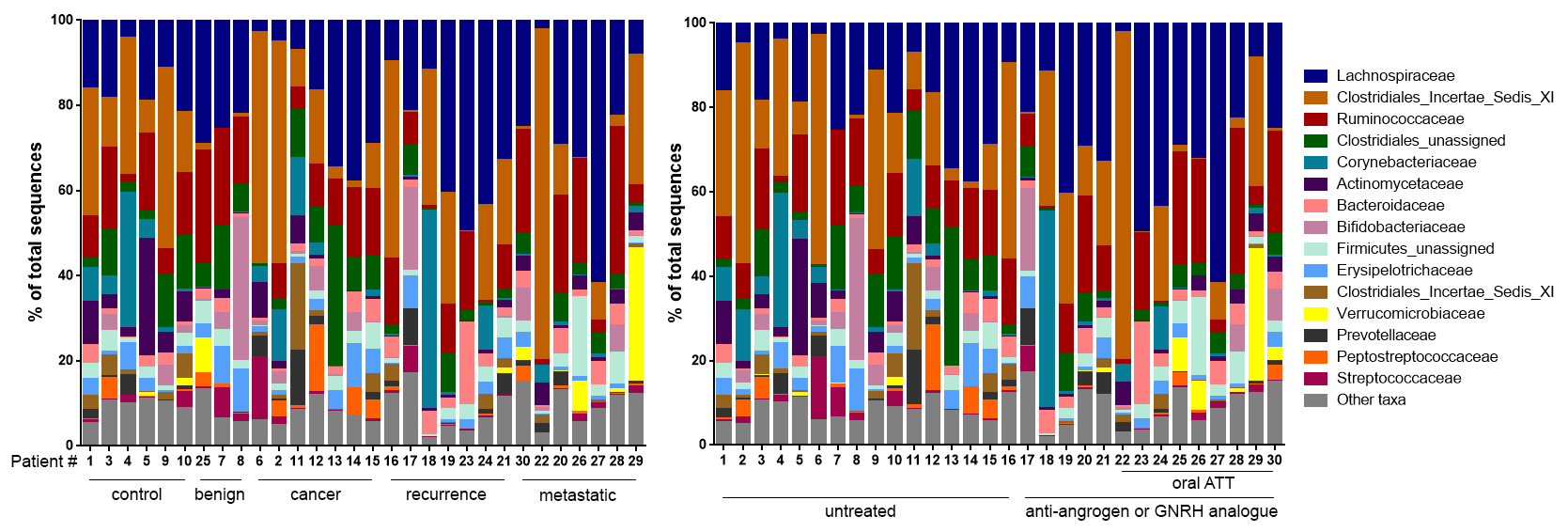

Supplement: Supplementary file 6 — Supplemental Figure S1 [file 41391_2018_61_MOESM6_ESM.tif]
